# Multielement Composition Analysis of Cicada as an Edible Insect and Dietary Risk Assessment

**DOI:** 10.3390/toxics13110916

**Published:** 2025-10-24

**Authors:** Guotao Ding, Mengyu Liu, Yanfei Fang, Peng Sun, Yonghong Han, Yingying Lian, Weihao Li

**Affiliations:** Handan Municipal Centre for Disease Prevention and Control, No. 581 Beicang Road (A), Handan 056008, China; liumengyu115@sina.com (M.L.); fangyanfei189@163.com (Y.F.); sunrise0603@163.com (P.S.); liguiying135@sina.com (Y.L.)

**Keywords:** cicada, entomophagy, multielement analysis, Hazard quotient (HQ), Hazard quotient (ICP-MS)

## Abstract

Cicadas are a traditional food in China. In this study, we performed multielement analysis on cicadas using Inductively Coupled Plasma–Mass Spectrometry (ICP-MS). In total, 40 cicada samples were collected in Hebei Province (China). Comparing the results of mushroom and vegetable samples selected as the standard foods among the eight food categories, which had the highest correlation with other food categories, there are 12 elements (Al, Mn, Zn, Cu, Cd, Ba, Se, V, As, Li, Pb and Co) in cicadas both higher than mushroom and vegetable samples, which a total of 25 elements were detected. The Principal Component Analysis (PCA), cluster analysis, and correlation analysis were used in the data analysis. HQ (Hazard quotient) value for Cd, As, Pb, and Al is 0.281 in males and is 0.324 in females. Concentrations of essential trace elements (Zn, Cu, and Se) in cicadas ranged from 1.7 to 101.9 times higher than those in mushrooms or vegetables. We analyzed in detail the two perspectives: one is the risk of potentially toxic elements, and the other is the enrichment of essential trace elements. In this study, we reveal that cicadas show a low risk of potentially toxic elements and high concentrations of essential trace elements, making them an edible insect.

## 1. Introduction

The history of entomophagy dates back at least 2000 years throughout the world, including Southeast Asia, Africa, Australia, and Latin America [1,2,3]. The first three questions for this study are: What insects are edible? Why eat insects? How to eat them? More than 2000 species of insects are consumed as food in the world, including beetles, caterpillars, bees, wasps, ants, grasshoppers, locusts, crickets, cicadas, leafhoppers, planthoppers, scale insects, true bugs, termites, dragonflies, flies, and other orders [4,5,6]. Food shortages have been a daunting and serious problem, as the global population continues to increase and individuals live longer [7]. Insects, as a food, contain high concentrations of protein, vitamins, essential trace elements, and easily digestible fatty acids [8,9,10]. Therefore, the consumption of insects can reduce the health damage caused by malnutrition in areas where food is scarce. Many researchers have investigated residents’ consumption intentions regarding the consumption of insects as a sustainable food [11]. Insects are often processed, such as roasting, frying, and boiling, before being eaten. These preparations can enhance the flavor and texture of insects [12]. After addressing the three questions, we show that the subject of investigation and assessment of multielement composition for edible insects is more important.

Increasing research has focused on the risk assessment of potentially toxic elements in food, such as cadmium (Cd), arsenic (As), lead (Pb), and aluminum (Al), with the development of industry and agriculture. Adverse effects of Cd include causing “itai-itai” disease and kidney disease [13,14]. Exposure to inorganic As may cause chronic toxicities and increase the risk of kidney, liver, and skin cancers [15]. Adverse effects of Pb include brain damage, high blood pressure, kidney damage, and cancer [16]. Regardless of the doses of Al, the toxicity attributed to Al is potentially harmful to the nervous system of humans [17]. The research, which is about dietary risk assessment in vegetables, crops, meats, and other food categories, has been carried out throughout the world, and high risk is reported in some areas [18,19,20,21].

The concentration of essential trace elements in food is an important indicator of the nutritional value of food. Zinc (Zn), copper (Cu), and Selenium (Se) are important for human health. Zn deficiency has been associated with the stunting of children’s lives and bodies, anemia, and susceptibility to infections [22,23]. The lack of Cu can increase the risk of cardiovascular diseases [24]. Se deficiency causes cardiomyopathy, known as Kashin disease, and causes an endemic osteoarthropathy, known as Kashin–Beck disease [25,26]. Eating foods rich in Zn, Cu, and Se can effectively reduce the risk of diseases caused by Zn, Cu, and Se deficiency. Therefore, foods rich in Zn, Cu, and Se are considered to have higher nutritional value.

In previous studies, we have analyzed multiple elements in fish, meat, dairy products, grains, vegetables, and mushrooms. After conducting a correlation analysis of the results, it was found that vegetable and mushroom samples had the highest correlation with other food categories. 32 mushroom samples and 34 vegetable samples were collected from 20 counties and districts of Hebei Province (China) and subjected to analysis. Therefore, cicadas were compared with vegetable and mushroom samples, and then the results of edible insects were analyzed according to the comparison results. The aim was to analyze the risk of potentially toxic elements and the content of essential elements. If the HQ of potentially toxic elements is high and the content of essential elements is low, it indicates that consuming cicadas would be meaningless in terms of element intake levels. Otherwise, it indicates that eating cicadas is relatively safe. The feasibility of eating cicadas was analyzed from two perspectives: the risk of potentially toxic elements and the rich essential trace elements.

## 2. Materials and Methods

### 2.1. Chemicals, Standards, and Instruments

Nitric acid 60% and hydrochloric acid 32% (high purity acids grade) are purchased from Merck Co., (Taufkirchen, Germany). Hydrogen peroxide 30% aqueous solution (ultra-Metal-oxide-semiconductor, CMOS grade) was obtained from Sinopharm Chemical Reagent Co., (Shanghai, China). Quality Control samples (QC samples), the chemical composition in Spinacia powder (GBW10015a), and the chemical composition in Scallop powder (GBW10024), were acquired from the Institute of Geophysical and Geochemical Exploration (Beijing, China). Multi-element Solution Certified Reference Material (GNM-M 259345), Internal Standard Mix, and Single Element Standard Certified Reference Material (K, Ca, Fe, Mg, Na) were acquired from GRINM GROUP (China General Research Institute for Nonferrous Metals, Beijing, China). NEXIONTM 350X ICP-MS and NEXION Setup Solution were purchased from PerkinElmer (Hopkinton, MA, USA). A High-Performance Microwave Digestion System was obtained from LabTech (Beijing, China). Precision balance, 0.1 mg readability, was acquired from METTLER TOLEDO (Zurich, Switzerland). An ultrapure water production system, whose model is Gradient A10, was purchased from Millipore (Taufkirchen, Germany).

### 2.2. Sample Preparation

In August, 40 cicada (*Cryptotympana atrata*) samples were collected from local markets in 20 counties and districts in Hebei Province (China). After the samples were sent to the laboratory, they were minced at low temperature and stored at −18 °C until analysis. All the samples were prepared separately. Powdered sample (approximately 0.2 g) was weighed in triplicate, transferred to digestion vessels with 8 mL of 60% nitric acid, soaked overnight, and then 2 mL of Hydrogen peroxide 30% aqueous solution was added. The samples were digested using the High-Performance Microwave Digestion System under the following conditions: 1. Rise from room temperature to 120 °C using 5 min, and retain 5 min. 2. Rise from 120 °C to 160 °C using 5 min, and retain 10 min. 3. Rise from 160 °C to 200 °C using 5 min, and retain 25 min. Subsequently, the digestion solution was evaporated less than 1 mL at 140 °C, diluted by ultrapure water to 10 mL, and analyzed by ICP-MS. The vegetable, mushroom and QC samples involved in the comparison in this paper were also treated in the same microwave digestion procedure. All the vessels were cleaned with 10% hydrochloric acid and then rinsed with ultra-pure water before use.

### 2.3. ICP-MS Condition

The torch alignment wizard and the detector voltage SmartTune wizard (Thermo Fisher Scientific, Waltham, MA, USA) were run before smart tuning was started using the NEXION setup solution (PerkinElmer). Plasma gas (argon) was set at 19 L/min, auxiliary gas (argon) at 1.2 L/min, and nebulizer gas (argon) at 1 L/min. The scan mode for all elements was peak hopping, and the dwell time for all elements was 50 ms per atomic mass unit (AMU). The radio frequency (RF) power was 1600 W. All elements were tested under the conditions described above.

### 2.4. Method Validation

According to the content of each element in the previously investigated food, 25 elements were divided into three concentration series. The only difference among the three groups is that the concentrations of the calibration curves are different. The high concentration group included 5 elements (K, Ca, Na, Mg, and Fe) with concentrations of 0.5, 1, 5, 10, 30, and 50 mg/L. The medium concentration group included 11 elements (Al, Mn, B, Li, Zn, Cu, Sr, Cd, Sn, Ba, and Rb), and the concentrations were 3, 5, 10, 30, 50, 100, 300, 500 μg/L, respectively. The low concentration group included 9 elements (V, Cr, Co, Ni, As, Se, Mo, Sb, and Pb), and the concentrations were 0.1, 0.5, 1.0, 3, 5, 10, 30, 50, 100 μg/L, respectively. Spiking concentrations of 5 μg/Kg and blank were tested 10 times.

MDL = 3 × standard deviation of 10 blank detections/sensitivity

MDL is the abbreviation of Method Detection Limit. The sensitivity was defined as the slope of the spiked sample and the blank sample [27]. The recoveries of 25 elements were performed by spiking at a concentration of 10 mg/L in the high concentration group, at a concentration of 50 μg/L in the medium concentration group, and at a concentration of 10 μg/L in the low concentration group. To determine the accuracy of the assay, one spinach powder QC sample and one scallop QC sample were inserted in each of the ten samples.

### 2.5. Dietary Risk Assessment

The dietary risk assessment of cicadas, an edible insect, was performed by Hazard Quotient (HQ).

EDI = C × IR × BW/R.

EDI is the estimated daily intake. C is the exposed concentration. In this study, the 95th percentile (P95) concentration is used in the calculation to make the assessment more rigorous. IR is the intake rates of edible insects, 133 Kg/100 people/Year [5]. BW is body weight. R is a coefficient to transform the unit from Kg/100 people/Year to Kg/1 people/week, and is 100 × 53.

Hazard Quotient (HQ) = EDI/PTWI

PTWI is the abbreviation for provisional tolerated weekly intake. PTWI of Cd is 5.8 μg/kg bw, PTWI of As is 15 μg/kg bw, PTWI of Pb is 25 μg/kg bw, and PTWI of Al is 2000 μg/kg bw [28].

The HQ is a very important concept in potentially toxic elements risk assessment. HQ less than or equal to 1 indicates that adverse effects are not likely to occur and thus can be considered to have negligible hazard.

### 2.6. Data Analysis

In order to understand the content of multiple elements in Cicadas, all 25 elements were arranged in ascending order according to the average concentration, and the elements were divided into three groups: concentration less than 1 (mg/Kg), concentration more than 1 and less than 10 (mg/Kg), and concentration more than 10 (mg/Kg). In this study, we compared the mean, median, and P95 values of each element concentration in Cicada samples, vegetable samples, and mushroom samples. Raw data on the concentrations of the 25 elements in cicadas, mushrooms, and vegetables were presented in the Supplementary Materials. The mean and median concentrations of 12 elements (Al, Mn, Zn, Cu, Cd, Ba, Se, V, As, Li, Pb, and Co) are both greater than mushroom and vegetable samples. Using the SPSS Statistics 27 software, we compared the correlation coefficients of each element to three food categories: cicadas, mushrooms, and vegetables. The Principal Component analysis (PCA) of all samples was performed according to element classification. The HQ of 4 potentially toxic elements (Cd, As, Pb, and Al) was used for dietary risk assessment, and the concentration of 3 essential elements (Zn, Cu, and Se) was analyzed by ratio with vegetables and mushrooms.

## 3. Result and Discussion

### 3.1. Calibration Curves, MDL, and Recoveries

**Figure 1:**
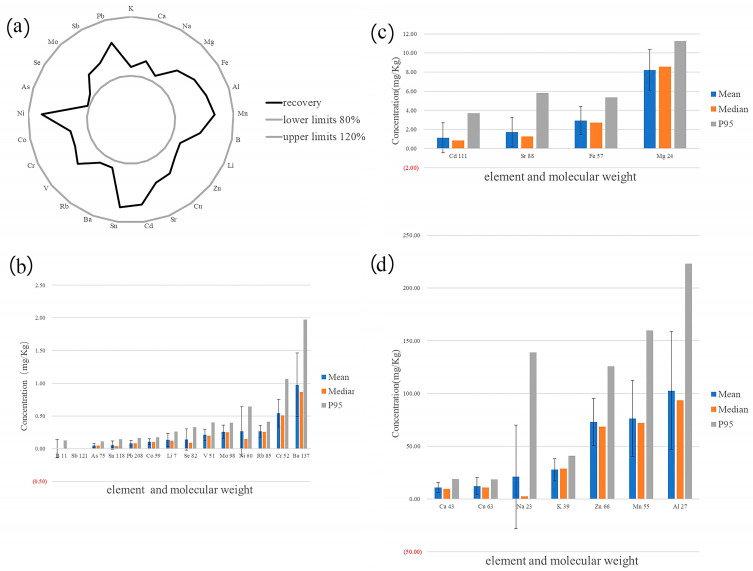
The recoveries for 25 elements and Concentrations in Cicadas. (**a**) The recoveries for 25 elements; (**b**) Concentrations below 1 mg/Kg in Cicadas; (**c**) Concentrations range from 1 mg/Kg to 10 mg/Kg in Cicadas; (**d**) Concentrations above 10 mg/Kg in Cicadas. The error line in the bar chart was the full sample concentrations’ standard deviation (SD).

The correlation coefficient R, the BEC (background equivalent concentration), and MDL for all elements are shown in Table 1. The correlation coefficients for 25 elements were more than 0.9990. The BEC for B, Zn, Al, and Li was 2.07 μg/L, 2.76 μg/L, 1.56 μg/L, and 1.47 μg/L, respectively. The MDL of this method is more than 1 μg/L for B, Zn, and Al. The detection rate of boron was only 12.5%. The average concentration of Zn and Al was 73.15 mg/Kg and 102.78 mg/Kg, respectively. The average concentration is much higher than that of BEC and MDL; therefore, BEC and MDL meet the detection requirements. The recoveries were shown in Figure 1a. The recoveries of all 25 elements were between 80% and 120%. The test results of QC samples were shown in Table 2. The results of triplicate measurements were consistent with the allowable error range of QC samples.

### 3.2. Multielements in Cicadas, Mushrooms, and Vegetables

The mean concentrations of 25 elements in cicada samples were sorted in ascending order. A total of 14 elements with average concentrations below 1 mg/Kg are shown in Figure 1b, including B, Sb, As, Sn, Pb, Co, Li, Se, V, Mo, Ni, Rb, Cr, and Ba. There are 4 elements, whose average concentrations range from 1 mg/Kg to 10 mg/Kg, were shown in Figure 1c, including Cd, Sr, Fe, and Mg. 7 elements with an average concentration above 10 mg/Kg are shown in Figure 1d, including Ca, Cu, Na, K, Zn, Mn, and Al. Raw data on the concentrations of the 25 elements in cicadas, mushrooms, and vegetables will be presented in the Supplementary Materials. We will compare the mean and median values in cicadas, mushrooms, and vegetables, and select elements with higher concentrations than mushrooms and vegetables regardless of the mean and median values. The mean and median values of 12 elements (Al, Mn, Zn, Cu, Cd, Ba, Se, V, As, Li, Pb, and Co) were higher than mushroom and vegetable samples.

**Figure 2:**
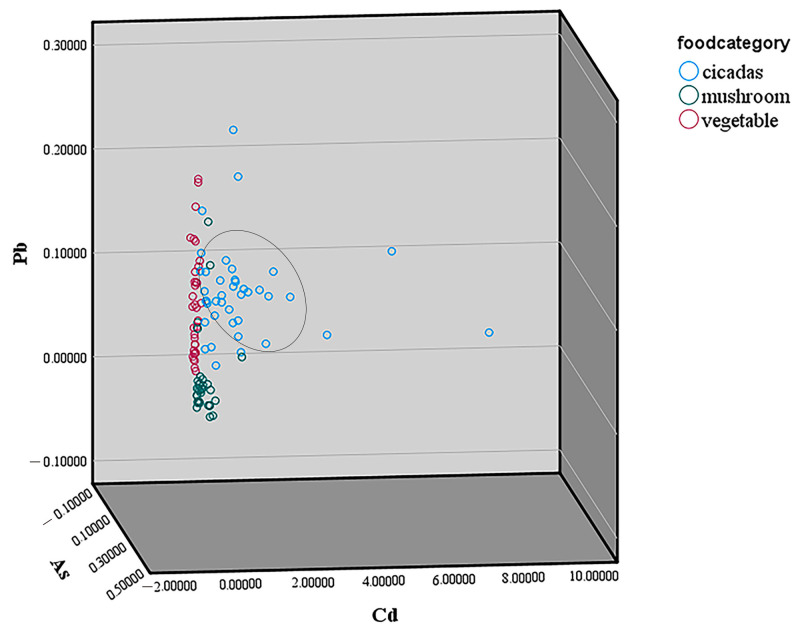
The cluster analysis of Pb, As, and Cd in three food categories. The points circled in the figure are derived from the clustering of cicadas.

For dimensionality reduction of complex datasets, we used PCA in this study. The first five PCs could account for 79.45% of the Cumulative Variance of Rotated Component Loadings. Among the four potentially toxic elements, Cd and Al in PC1 extract 20.62% of the total variance, As in PC2 extract 19.20% of the total variance, and Pb in PC3 extract 17.83% of the total variance. Among the three essential elements, Zn and Cu in PC1, as well as Se in PC4, account for 12.89% of the total variance. The dietary risk assessment was carried out after applying the weight function to all the data. The cluster analysis of Pb, As, and Cd in three food categories is shown in Figure 2. The concentration of Cd in cicada was significantly higher than that in mushrooms and in vegetables.

**Figure 3:**
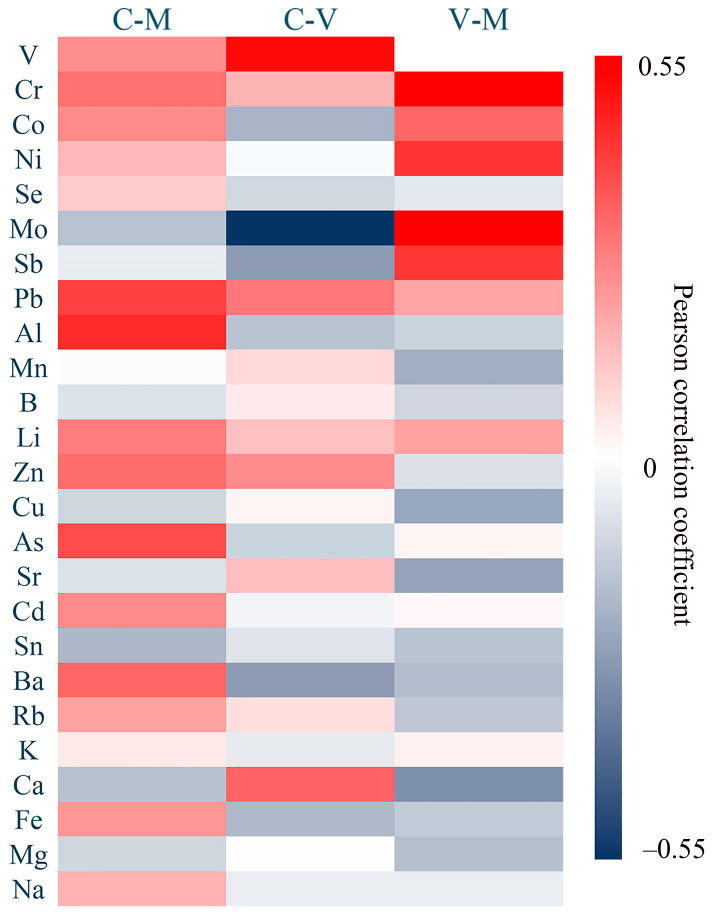
The Heat map of pairwise correlation in three food categories for each element. C-M means the correlation between cicadas and mushrooms; C-V means the correlation between cicadas and vegetables; and M-V means the correlation between mushrooms and vegetables.

The pairwise correlation for each element across three food categories is shown in Figure 3. The Pearson correlation coefficient for V between cicadas and vegetables was 0.366 (*p* < 0.05). The Pearson correlation coefficient for Cr between mushrooms and vegetables was 0.386 (*p* < 0.05). The Pearson correlation coefficient for Mo between cicadas and vegetables was −0.449 (*p* < 0.01), and between mushrooms and vegetables was 0.394 (*p* < 0.05).

### 3.3. Dietary Risk Assessment

In this paper, 4 potentially toxic elements (Cd, As, Pb, and Al) are calculated using HQ to assess dietary risk. The HQ was shown in Table 3. The average concentration of Cd in Cicadas was 30.1 times higher than in mushrooms, and 53.0 times higher than in vegetables. The median concentration of Cd in Cicadas was 40.1 times higher than in mushrooms, and 43.0 times higher than in vegetables. Due to the high concentration of Cd, eliminating the interference of Cd to make the test results accurate is one of the key points of this study. ^96^Mo^16^O^+^ and ^114^Sn interfere with ^111^Cd^+^ and ^114^Cd^+^, respectively [29]. The mean and median concentration of Mo were 0.26 mg/Kg and 0.25 mg/Kg. The mean and median concentration of Sn were 0.05 mg/Kg and 0.04 mg/Kg, respectively. To avoid the Mo element interfering with the results, ^144^Cd^+^ was selected for detection while using the correction equation.

The correction equation:

^114^Cd^+^ = counts per second (CPS) at 114 AMU − (0.66%/24.22%) × ^118^Sn

The abundance of ^114^Sn was 0.66% and that of ^118^Sn was 24.22%.

The concentration of Cd in this paper is the result after correction by the correction equation.

### 3.4. Concentration of 3 Essential Elements

Concentrations of essential trace elements (Zn, Cu, and Se) in cicadas, mushrooms, and vegetables are shown in Table 4. In cicadas, the concentration of Zn was highest, with a mean value of 73.15 mg/Kg and a median value of 68.59 mg/Kg. In mushrooms, the concentration of Zn was highest too, with a mean value of 10.37 mg/Kg and a median value of 10.27 mg/Kg. All samples (cicadas, mushrooms, and vegetables) showed higher Zn concentration than Cu concentration and higher Cu concentration than Se concentration. The mean and median Cu concentrations in cicadas were divided from mushrooms and vegetables, and the ratio was greater than that of Zn and Se. The ratio of mean and median (Cicada/Mushroom) was 9.1 and 10.7, respectively. The ratio of mean and median (Cicada/Vegetable) was 69.0 and 101.9, respectively.

## 4. Conclusions

Consuming insects as food could solve many of the problems caused by malnutrition, mostly in developing countries. Insects contain high concentrations of protein, vitamins, essential trace elements, and easily digestible fatty acids. However, dietary risk assessment for insect consumption has not been studied extensively.

In this study, we performed multielement analysis of cicadas using Inductively Coupled Plasma–Mass Spectrometry (ICP-MS). 12 elements were found to be higher than both the mushroom and the vegetable from the 25 elements detected. Of the 12 elements, the dietary risk of 4 potentially toxic elements was assessed, and the content of 3 essential elements was analyzed. The results indicate that HQ value for Cd, As, Pb, and Al in males and females was less than 1.0. An HQ of less than 1 indicates that adverse effects are not likely to occur. Thus, the 4 selected potentially toxic elements can be considered to have a negligible hazard. The concentrations of essential trace elements (Zn, Cu, and Se) in cicadas ranged from 1.7 to 101.9 times higher than those in mushrooms or vegetables. Of course, the PCA, cluster analysis, and correlation analysis also found some information that caught our attention. Pearson correlation analysis indicated that the concentrations of vanadium (V) was positively correlated with the vegetables 24 elements (except Li, Zn and Sn) in vegetables and cicadas. Does the element V exhibit any phenomenon of antagonistic migration in plants and insects compared to other elements? And whether the correlation of elements V, Cr, and Mo in different food categories is related to biological cometabolism. This is the next scientific topic that we are going to study. At the end of the research, we declare that consuming cicadas is relatively safe in two ways. One aspect is the lower risk of potentially toxic elements, and the other aspect is the higher concentration of essential trace elements.

## Figures and Tables

**Table 1:** Analytical performances for 25 elements.

Element	Mass	R	BEC (μg/L)	MDL (μg/L)
K	39	0.9994	0.152	0.017
Ca	43	0.9991	0.104	0.142
Na	23	0.9999	0.053	0.149
Mg	24	0.9998	0.059	0.025
Fe	57	0.9996	0.043	0.017
Al	27	0.9993	1.560	1.052
Mn	55	0.9994	0.448	0.026
B	11	0.9992	2.072	1.505
Li	7	0.9998	1.466	0.325
Zn	66	0.9999	2.760	1.414
Cu	63	0.9997	0.621	0.194
Sr	88	0.9995	0.454	0.150
Cd	114	0.9999	0.142	0.017
Sn	118	0.9991	0.721	0.145
Ba	137	0.9990	0.278	0.521
Rb	85	0.9997	0.289	0.063
V	51	0.9994	0.121	0.040
Cr	52	0.9991	0.510	0.381
Co	59	0.9997	0.174	0.046
Ni	60	0.9999	0.363	0.164
As	75	0.9999	0.926	0.273
Se	82	0.9993	0.994	0.694
Mo	98	0.9997	0.132	0.046
Sb	121	0.9998	0.157	0.013
Pb	208	0.9999	0.360	0.170

**Table 2:** The concentration of 25 elements in the QC sample.

Element	Spinacia Powder		Element	Scallop Powder	
	Certified Concentration	Measured Concentration		Certified Concentration	Measured Concentration
K *	4.0 ± 0.2	4.0	K *	1.15 ± 0.06	1.01
Ca *	0.88 ± 0.09	0.79	Ca *	0.075 ± 0.009	0.075
Na	1.80 ± 0.07	1.85	Na *	0.46 ± 0.04	0.45
Mg *	0.99 ± 0.07	1.05	Mg *	0.174 ± 0.006	0.169
Fe	0.093 ± 0.007	0.090	Fe	41 ± 5	41
Al *	0.16 ± 0.03	0.15	Al *	0.0156 ± 0.0027	0.0161
Mn	80 ± 4	83	Mn	19.2 ± 1.2	19.8
B	30.6 ± 4.2	33.4	B	12 ± 1	13
Li	3.8 ± 0.5	4.2	Li	0.13 ± 0.02	0.12
Zn	42 ± 4	44	Zn	75 ± 3	77
Cu	10.4 ± 0.8	11.0	Cu	1.34 ± 0.18	1.28
Sr	120 ± 7	124	Sr	6.5 ± 0.4	6.2
Cd **	190 ± 20	182	Cd	1.06 ± 0.1	1.12
Sn	0.062 ± 0.007	0.058	Sn	0.13 ± 0.004	0.13
Ba	10.9 ± 1.7	10.1	Ba	0.62 ± 0.06	0.59
Rb	31 ± 2	31	Rb	5.1 ± 0.3	4.9
V	2 ± 0.01	2.01	V	0.36 ± 0.1	0.44
Cr	9.0 ± 1.3	10.01	Cr	0.28 ± 0.07	0.30
Co	0.49 ± 0.03	0.48	Co	0.047 ± 0.006	0.047
Ni	1.9 ± 0.2	1.8	Ni	0.29 ± 0.08	0.21
As	0.54 ± 0.06	0.49	As	3.6 ± 0.6	4.0
Se	0.09 ± 0.01	0.09	Se	1.5 ± 0.3	1.7
Mo	0.65 ± 0.08	0.67	Mo	0.066 ± 0.016	0.070
Sb	0.038 ± 0.007	0.035	Sb	0.014 ± 0.002	0.013
Pb	1.07 ± 0.09	0.99	Pb	0.12 ± 0.007	0.13

The concentrations of elements with * were 10 g/Kg. The concentration of the element with ** was μg/Kg. The concentrations of elements without any maker were mg/Kg.

**Table 3:** HQ for 4 elements in cicadas.

Elements	Gender	P95(mg/Kg)	IR (Kg/100 People/Year)	BW (Kg)	PTWI [mg/(kg bw Weeks)]	HQ
**Cd**	Male	3.45	133	66.2	0.0058	0.2310
	Female	3.45	133	57.3	0.0058	0.2669
**As**	Male	0.13	133	66.2	0.015	0.0033
	Female	0.13	133	57.3	0.015	0.0037
**Pb**	Male	0.16	133	66.2	0.025	0.0024
	Female	0.16	133	57.3	0.025	0.0028
**Al**	Male	233.03	133	66.2	2	0.0442
	Female	233.03	133	57.3	2	0.0510

**Table 4:** Concentrations and ratios of essential trace elements (Zn, Cu, and Se) in cicadas, mushrooms, and vegetables.

Elements	Cicada		Mushroom				Vegetable			
	Mean (mg/Kg)	Median (mg/Kg)	Mean (mg/Kg)	Ratio of Mean (Cicada/Mushroom)	Median (mg/Kg)	Ratio of Median (Cicada/Mushroom)	Mean (mg/Kg)	Ratio of Mean (Cicada/Vegetable)	Median (mg/Kg)	Ratio of Median (Cicada/Vegetable)
**Zn**	73.149	68.591	10.368	7.0554	10.270	6.6786	2.276	32.139	2.006	34.193
**Cu**	12.204	11.017	1.337	9.128	1.031	10.684	0.177	68.992	0.108	101.897
**Se**	0.140	0.091	0.057	2.475	0.052	1.738	0.004	31.846	--	*

-- indicate less than the MDL (0.000694 mg/Kg); * indicate the absence of a ratio result.

## Data Availability

The authors have permission to share data.

## Supplementary Materials

The following supporting information can be downloaded at: https://www.mdpi.com/article/10.3390/toxics13110916/s1. Table S1: The concentrations of 25 elements and in Cicadas, mushrooms, and vegetables.
